# Phononic topological insulators based on six-petal holey silicon structures

**DOI:** 10.1038/s41598-018-38387-5

**Published:** 2019-02-12

**Authors:** Ziqi Yu, Zongqing Ren, Jaeho Lee

**Affiliations:** Mechanical and Aerospace Engineering, University of California, Irvine, Irvine, 92697 USA

## Abstract

Since the discovery of the Quantum Spin Hall Effect, electronic and photonic topological insulators have made substantial progress, but phononic topological insulators in solids have received relatively little attention due to challenges in realizing topological states without spin-like degrees of freedom and with transverse phonon polarizations. Here we present a holey silicon-based topological insulator design, in which simple geometric control enables topologically protected in-plane elastic wave propagation up to GHz ranges with a submicron periodicity. By integrating a hexagonal lattice of six small holes with one central large hole and by creating a hexagonal lattice by themselves, our design induces zone folding to form a double Dirac cone. Based on the hole dimensions, breaking the discrete translational symmetry allows the six-petal holey silicon to achieve the topological phase transition, yielding two topologically distinct phononic crystals. Our numerical simulations confirm inverted band structures and demonstrate backscattering-immune elastic wave transmissions through defects including a cavity, a disorder, and sharp bends. Our design also offers robustness against geometric errors and potential fabrication issues, which shows up to 90% transmission of elastic waves even with 6% under-sized or 11% over-sized holes. These findings provide a detailed understanding of the relationship between geometry and topological properties and pave the way for developing future phononic circuits.

## Introduction

The concept of topology^1,2^, or conserved properties under continuous deformation, has attracted much interest in recent years of condensed matter physics, since the discoveries of the Quantum Hall effect and Quantum Spin Hall effect^3–5^. The topological states have been first studied in electronic systems, and topological insulators are characterized by unique attributes of insulating bulk bands and conducting edge bands. These conducting bands are robust and protected by non-trivial topological states to support unidirectional propagation at the boundary with no backscattering even in the presence of defects, offering unmatched tolerance and unprecedented transport capabilities. While most of the interest and efforts have been in topological electronics^1,2^ and photonics^6,7^ due to the intrinsic spinning nature of the particles and the ease of breaking the time-reversal symmetry by the external magnetic field. Topological insulators based on bosonic systems have not been explored as much because of the lack of spin-like states. Moreover, the low group velocity, high density of states, and significantly dissimilar acoustic impedance between common materials induce backscattering, resulting in difficulties in achieving defect-immune wave propagations. Recently, investigations of topological insulators in mechanical^8,9^, acoustic^10–13^, and elastic^14–21^ systems have shown promising progress in tackling the challenges. The breaking of time-reversal symmetry was done by circulating fluid flow in the background^10,22^, external optomechanical excitation^23^, and periodically arranged local resonators^21^. Most success made on phononic systems have targeted on realizing symmetry protected edge states for acoustic waves possessing only one longitudinal polarization. Realizing helical edge states in elastic waves remains outstandingly challenging due to the fact that its three available polarizations (one longitudinal and two transverse) can easily be mixed at most solid interfaces precluding the formation of two degenerate states characterized by Dirac dispersions^24,25^. And most passive elastic materials generally conserve time-reversal symmetry, further impeding the fulfillment of chiral edge states in elastic systems^24–26^. Recent studies overcame these problems by utilizing chiral interlayer coupling to break the inversion symmetry^12^ or by emulating the Quantum Valley Hall effect to support edge states in artificially engineered elastic structures. The latter concept allows reduced geometrical complexities and can be extended to photonic^27^, acoustic^28^, and elastic systems^29^. The elastic analogue of the Quantum Valley Hall effect^29^, has been numerically demonstrated by periodic structures with edge states within the bulk band gaps. Similar to the acoustic topological system realized by a double Dirac cone^30^, a recent theoretical study has demonstrated an elastic topological system with a double Dirac-cone by using subwavelength meta-structures^24^. Two recent studies demonstrated a snowflake porous structure to topologically guide the elastic wave at GHz ranges making it favorable for applications of phononic circuits^14,31^. Other studies show the possibility of realizing topological insulator by using perforated holes to enable elastic pseudospin transport^19^ and spiral-shaped pores to guide the flexural waves^32^. While there have been notable achievements in theoretical ends, there have been limited studies for elastic topological insulators especially in phonon frequency regimes of GHz and beyond and using a platform of silicon, which offers significant compatibility to semiconductor devices and fabrication feasibility. In this paper, we develop a novel design of elastic wave topological insulator based on six-petal holey silicon nanostructures which supports topologically protected wave propagation at frequencies up to GHz ranges when the unit cell periodicity reaches submicron scales even in the presence of geometric defects and potential fabrication issues. The circular pore shape provides higher tolerance to rounding effect in fabrication processes, making it competitive and desirable for the application of monolithic phononic circuits for information processing. The design also offers scalability from low- to high-frequency based on the periodicity, yielding the smallest neck size of ~20 nm in our current simulations and has been experimentally achieved in previous holey silicon nanostructure for thermal characterization^33^, opening up possibilities of feasible fabrication and experimental exploration.

## Results

### Design of six-petal holey silicon topological insulators

**Figure 1:**
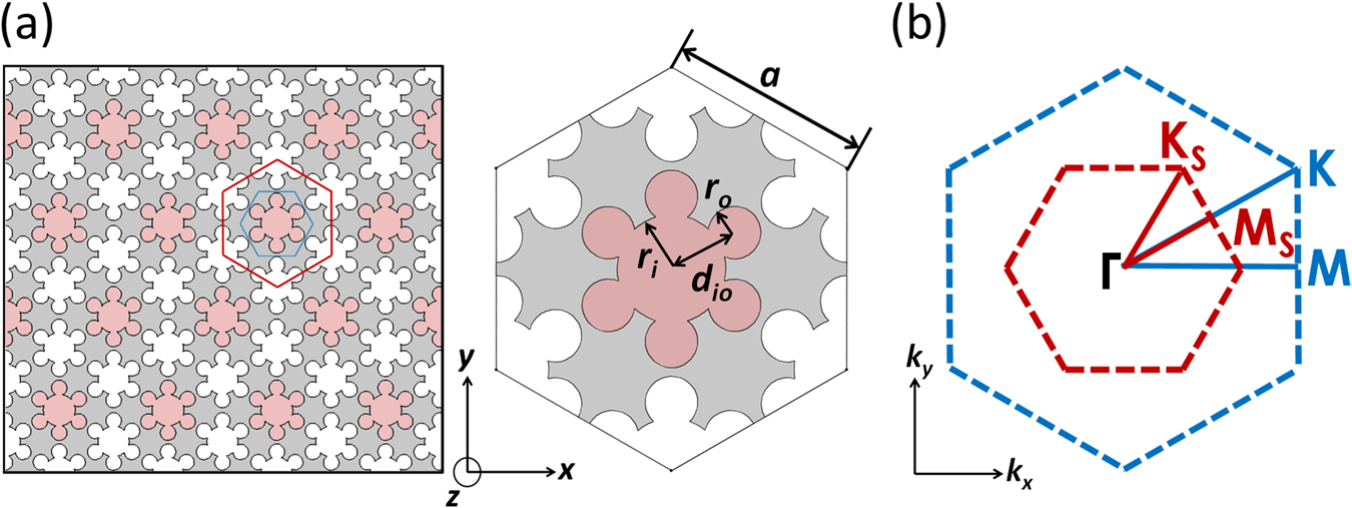
(**a**) Left: Schematics of the six-petal holey silicon in a hexagonal lattice. Right: The original unit cell containing a single six-petal pore (light-red filled) and the enlarged unit cell (gray filled) with the original one surrounded by one-third of each its six neighboring pores in real space. Each six-petal pore is defined by dimensional parameters (***r***_***i***_, ***r***_***o***_, ***d***_***io***_**)**. The thickness of the film is kept at 100 nm and the periodicity *a* is 866 nm. The close and open of the bandgap is realized by manipulating ***r***_***o***_ and ***d***_***io***_, namely, modifying **Δ*r***_***o***_ and **Δ*d***_***io***_. When **Δ*r***_***o***_ and **Δ*d***_***io***_ are zeros, the original hexagonal lattice has a discrete translational symmetry and we expect a double Dirac cone in the dispersion curve. When they are not zeros, the translational symmetry will be broken, and we expect the double Dirac cone to be replaced by band gaps in the dispersion curves. (**b**) Schematic of the first Brillouin zone for the original and enlarged unit cells in reciprocal space. The symmetry line for the original unit cell is Γ-M-K-Γ whereas the folded symmetry line for the enlarged unit cell is Γ-M_S_-K_S_- Γ.

The designed structure is a planar quasi-two-dimensional phononic crystal with a hexagonal lattice of vacuum six-petal-shaped pores perforated in a silicon slab (Fig. 1(a), left). Each pore consists of a larger circle in the center surrounded equidistantly by six smaller circles (Fig. 1(a), right). The radii of the central and corner circles are ***r***_***i***_ and ***r***_***o***_, respectively, and their center-to-center distance ***d***_***io***_ follows ***d***_***io***_ < ***r***_***i***_ + ***r***_***o***_. Previous studies on snowflake-hole-based phononic waveguides had reported the edge-rounding effect and sizing errors in the fabrication process^34,35^. The rounding of sharp corners may dramatically change the mechanical response leading to discrepancies between numerical simulations and experimental data^36^. Our design of six-petal pore significantly mitigates the negative effect from such fabrication imperfection by incorporating circles as the building block. Porous structures with circular features have been widely employed in various photonic and electronic systems. While noncircular or nonconventional geometries may provide better topological properties when fabrication inaccuracy is precisely controlled, our six-petal design offers excellent solutions against a wide range of uncertainties and high transmission via robust optimization, which is a unique approach fundamentally different from deterministic optimization. Our design opens up new pathways of achieving phononic topological insulators based on conventional platform having circular holes.

### Band folding and band inversion

**Figure 2:**
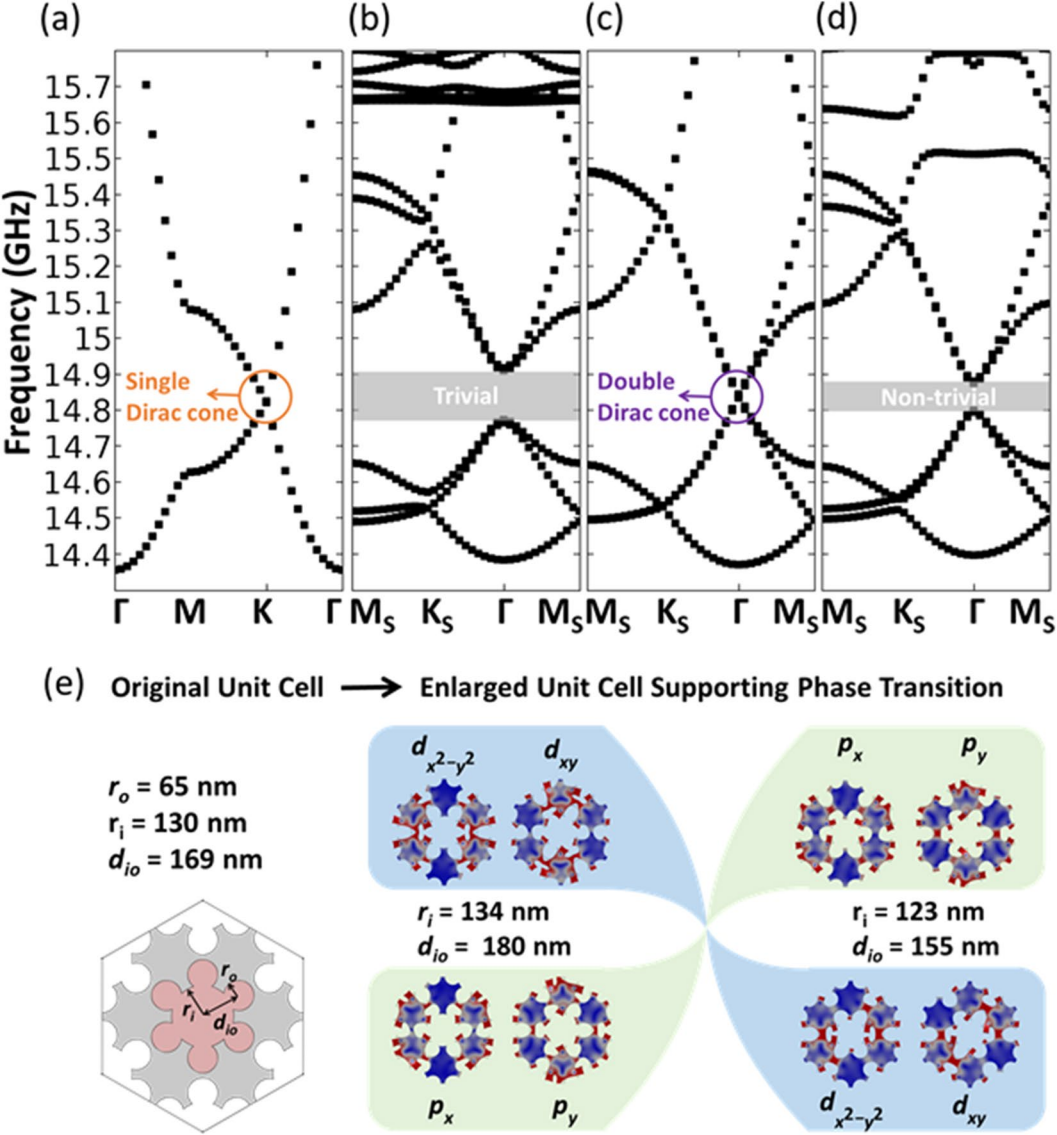
Elastic topological insulator design based on six-petal holey silicon nanostructures, which supports the required band inversion process at GHz ranges and offers high tolerance to processing defects. The dimensions of the enlarged unit cell are shown where we keep ***r***_***o***_ the same and change ***r***_***i***_ and ***d***_***io***_. (**a**) Band structure of the original unit cell and the corresponding single Dirac cone at the point ***K***. (**b**–**d**) Band structures of the enlarged unit cell and the corresponding topological phase transition from the zone-folding-induced double Dirac cone to bandgaps by modifying the geometry. (**e**) The quadruples (*d*_*x*2*−y*2_ and *d*_*xy*_) are found at higher frequencies whereas dipoles (*p*_*x*_ and *p*_*y*_) are found at lower frequencies, which are trivial. When the band inversion occurs, the quadruples move to lower frequencies while the dipoles move to higher frequencies, which are considered non-trivial. The lattice constant ***a*** is kept at 866 nm. In the simulation, we consider the silicon with a Young’s modulus of 170 GPa, mass density of 2329 kg-m^−3^, and Poisson’s ratio of 0.28.

As the first step to designing a topological insulator, we create a single Dirac cone for in-plane elastic waves, whose existence has been demonstrated previously for the graphene-like hexagonal-lattice^25,32^. We choose a hexagonal unit cell containing a single six-petal pore, exhibiting a *C*_6_ symmetry (blue in Fig. 1(a)). To compute the dispersion properties of the unit cell, we solve the elastic wave equation for six-petal holey silicon using the finite element method in COMSOL Multiphysics with Floquet periodic boundary conditions. By optimizing the geometrical parameters to be (***r***_***i***_, ***r***_***o***_, ***d***_***io***_) = (65, 130, 169) nm, we can obtain a Dirac cone at *f* = 14.83 GHz (Fig. 2(a)). A thickness of 100 nm for the unit cell is selected to achieve a complete band gap for in-plane waves around the frequency *f*. To construct a double Dirac cone, we now consider an enlarged unit cell (red in Fig. 1(a)) with the original unit cell encircled by one-third of each of its six neighbors. This folds the original first Brillouin zone (1BZ) (blue in Fig. 1(b)) by a factor of 1/√3 and forms a two-fold degenerate Dirac cone (Fig. 2(c)) at the ***Γ*** point by mapping the Dirac cone at ***K*** in the original 1BZ to the new one. To realize the band inversion, we remain the radii of all the one-third pores (***r***_***o***_) in the enlarged unit cell as they are and modify ***r***_***i***_ and ***d***_***io***_. The breaking of the discrete translational symmetry, originally characterized by the lattice constant *a* = 500 nm, makes the enlarged unit cell be the smallest repeating cell with a pair of lattice vectors $\mathop{{a}_{1}}\limits^{\rightharpoonup }$ and $\mathop{{a}_{2}}\limits^{\rightharpoonup }$ and a larger lattice constant of 500√3 nm (866 nm). Based on the geometrical parameters shown in Fig. 2(e) for the enlarged unit cell, we obtain two topologically protected band gaps at the ***Γ*** point in the dispersion curves for two phononic crystals (PnCs) (Fig. 2(b,d)), with inverted degenerate modes at both gap edges illustrated by mechanical displacement (Fig. 2(e)). The quadruples (${d}_{{x}^{2}-{y}^{2}}$ and ${d}_{xy}$) appear at the high frequency in the trivial PnC (Fig. 2(b)), whereas they move to the low frequency in the non-trivial PnC (Fig. 2(d)). We notice that the neck size, which is the smallest channel size between adjacent pores, reaches ~20 nm in the trivial PnC, which may pose challenges to potential experimental demonstration requiring high-frequency (i.e., GHz ranges) mechanical response with high-fidelity. The novel design of six-petal holey silicon allows the topologically protected edge state to scale from low to high frequencies via geometric control, which enables topological insulators to operate over a wide range (See Supplementary Information Note 1). Piezo-electric transducers have been used to excite MHz-range elastic waves in out-of-plane^37,38^. For in-plane elastic waves considered in our simulation, interdigital transducer could be a practical candidate. The simulation results presented in the current work would still be applicable when experimental conditions are met and realized in the future.

### Emergence of topological states

**Figure 3:**
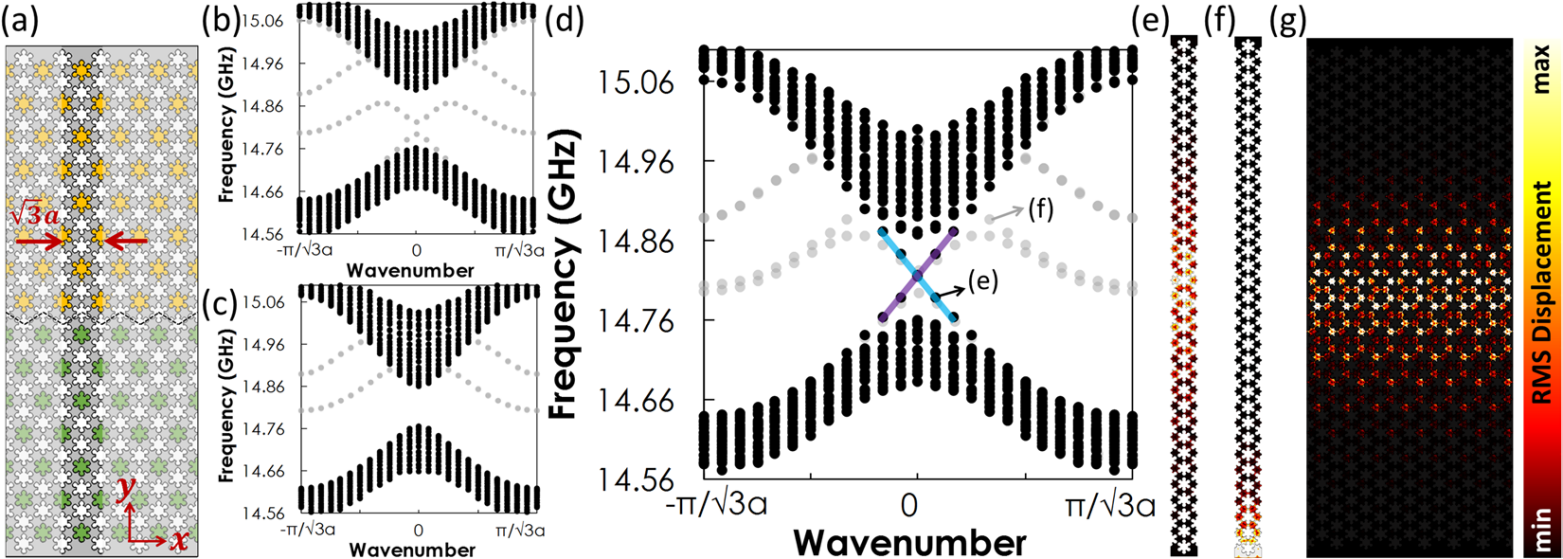
(**a**) The supercell used for computing the bulk band structure which comprises trivial (yellow) and non-trivial (green) PnCs separated by an interface represented by the black dashed line. The depicted supercell contains 11 enlarged unit cells in total with 5 non-trivial ones on top of and 6 trivial ones on bottom of the interface. (**b**) Projected bulk band structure of the trivial PnC. The extra bands (light-gray) inside the bulk bandgap are localized on top and bottom boundaries (**f**). (**c**) Projected bulk band structure of the non-trivial PnC. (**d**) Projected bulk band structure of a supercell. Edge bands supporting the topologically protected elastic wave transport and their crossing at the point ***Γ*** is shown by the blue and purple lines. Two representative mode shapes associated with (**e**) the edge bands and (**f**) bands at physical boundaries. The latter bands arise from the breakage of symmetry on top and bottom physical boundaries due to the truncated simulation domain and they are not symmetry-protected. (**g**) Topologically protected edge state along the domain wall formed between two topologically distinct PnCs. The edge state is highly localized around the domain wall, with a penetration depth into the bulk which decays fast.

At the interface between two topologically distinct domains, we expect to find the topologically protected edge states^1,14,16^. By connecting the trivial and non-trivial PnCs discussed above to form an interface, we demonstrate the existence of a topological state. We consider a supercell which is periodic along the *x* direction and finite in the *y* direction (Fig. 3(a)). We first calculate the dispersion curves for the supercells where the topologically identical PnCs are present on both sides of the interface. In Fig. 3(b,c), we can see the complete band gaps appearing in the bulk band structure except for bands (light-gray) confined at physical boundaries partially traversing the band gaps. They emerge due to the broken symmetry at the boundaries of the finite domain, which are neither related to the topological state at the interface nor protected by any symmetry^14^. When we have trivial and non-trivial PnCs on each side, the above band gaps are replaced by two crossing straight lines (Fig. 3(d)), indicating the emerge of topologically protected edge states highly localized at the interface (Fig. 3(e)). The bands confined at physical boundaries can also be observed, as displayed in Fig. 3(f). We further calculate the root-mean-square (rms) displacement of a system comprising of 6 abovementioned supercells placed adjacent to each other horizontally, which is shown in Fig. 3(g). The elastic wave excited at a frequency of 14.83 GHz, which is within the range of the bulk bandgap (Fig. 3(d)), propagates robustly from the left to the right demonstrating the topological protection. We notice that the elastic wave also penetrates into the bulk region but decays quickly, and the similar penetration has been observed in the previous study^14^.

### Robust transmission against geometrical defects

**Figure 4:**
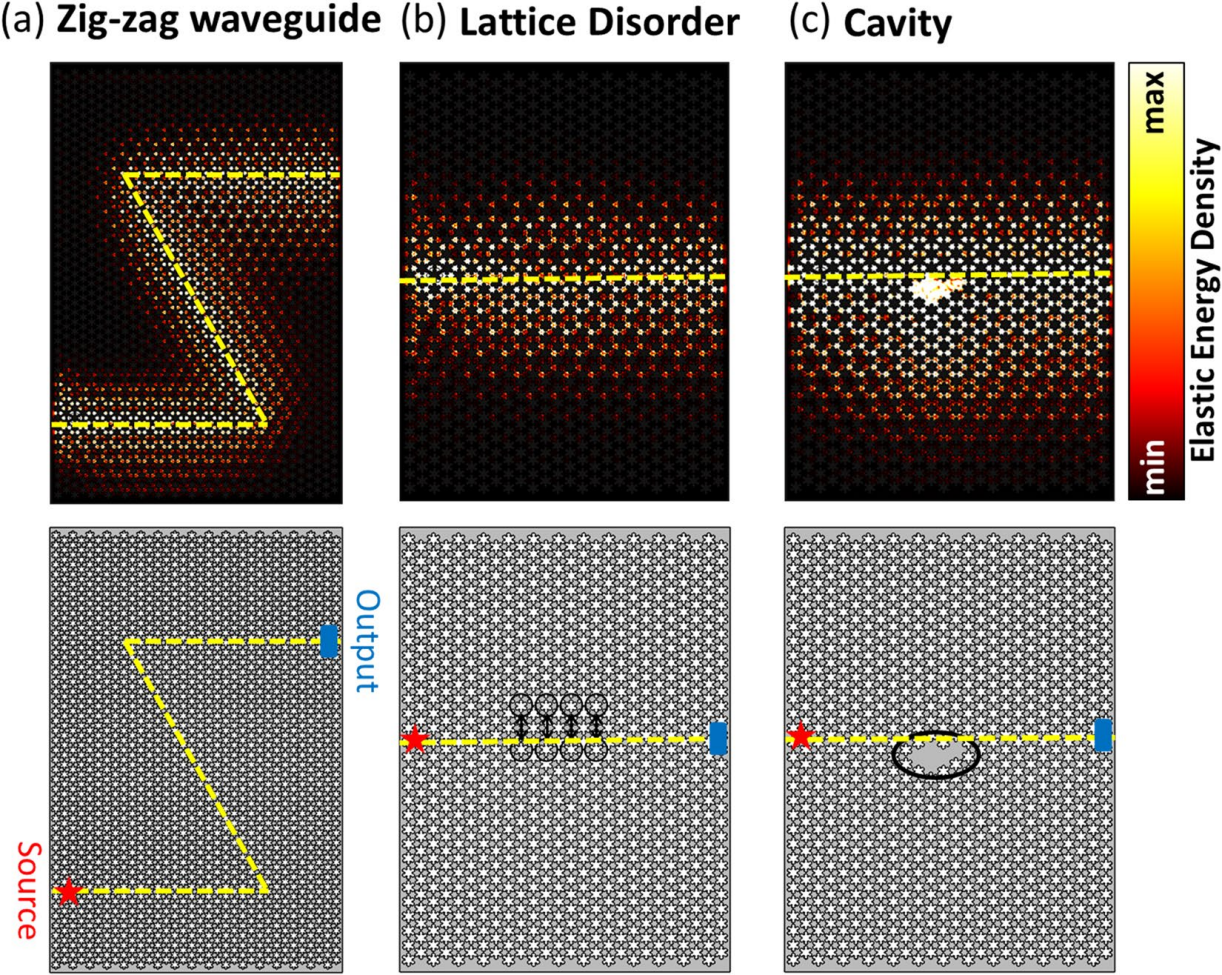
Topologically protected elastic wave transmission in (**a**) a zigzag waveguide, (**b**) near the interface with a lattice disorder, and (**c**) a cavity. The normalized elastic energy density shows high elastic wave transmission from left to right of the domain in all three cases (upper panel). We compute the transmission as the ratio between the elastic energy density at the source and the output. The quantified transmissions read 90%, 88%, and 90% for the zig-zag domain wall, lattice disorder, and cavity cases. As a comparison, we also simulate ordinary phononic waveguides with similar defects and the results indicate dramatically inhibited elastic wave propagation (transmission values are all less than 10%), which is in a distinct contrast to the high transmissions achieved by the topological insulators, due to the elastic resonance at the cavity and lattice disorder and backscattering at the sharp bends. The schematics depicting the zigzag interface in the waveguide, lattice disorders, and the cavity are displayed in the lower panel.

As the most striking characteristic of topological insulators, the interface between trivial and non-trivial PnCs supports robust elastic wave propagation even in the presence of non-spin-mixing defects^24,30,39^. As the first case, we introduce a zigzag domain wall functioning as a waveguide with two sharp bends of the angles of 60°, and then we evaluate cases including a lattice disorder formed by exchanging trivial and non-trivial unit cells across the interface and a cavity formed by filling several six-petal pores^19^, schematics of all three cases are depicted in Fig. 4 (lower panel). To avoid any leakage of elastic energy through physical boundaries, we apply low-reflection boundary conditions surrounding the simulation domain. We apply point harmonic excitations on three points in a unit cell separated by a length of *a* and having a phase delay of $\frac{2\pi }{3}$ between each two at the frequency of 14.83 GHz^16,18,23,31^. Similar multi-point excitation with carefully engineered amplitudes and phases can launch one-way elastic waves, which may favor applications that directional control is desired (See Supplementary Information Note 4). We then calculate the elastic energy density and show its distribution. The transmission of the elastic wave is quantified by taking the ratio of elastic energy densities at the source and the output. For the zig-zag waveguide case in Fig. 4(a), the elastic wave excited at the source is able to circumvent the sharp bends and arrive at the output with a transmission of 90% with no obvious backscattering. For the cases of lattice disorder and cavity (Fig. 4(b,c)), the elastic wave incident from the source maintains high transmission values of 88% and 90%, respectively, against these defects. The elastic wave propagation in all three cases localized closely in the vicinity of the interface and decays quickly into the bulk, indicating the insulating nature of the bulk region. In contrast, the results for the ordinary phononic waveguides with similar defects are drastically different (See Supplementary Information Note 2). We can clearly observe the occurrence of strong elastic resonances when the elastic wave runs into the cavity and lattice disorder, whereas the sharp bends along the zigzag domain wall inhibits significantly the elastic wave propagation by backscattering, resulting in a dramatic decrease of transmission down to less than 10%. The elastic transmission supported by the topologically protected edge state at the domain wall should theoretically equal to 100%, while our simulation results show some losses. This might be due to the limited simulation domain size we used to keep the computational effort manageable.

### Robust transmission against potential fabrication errors

**Figure 5:**
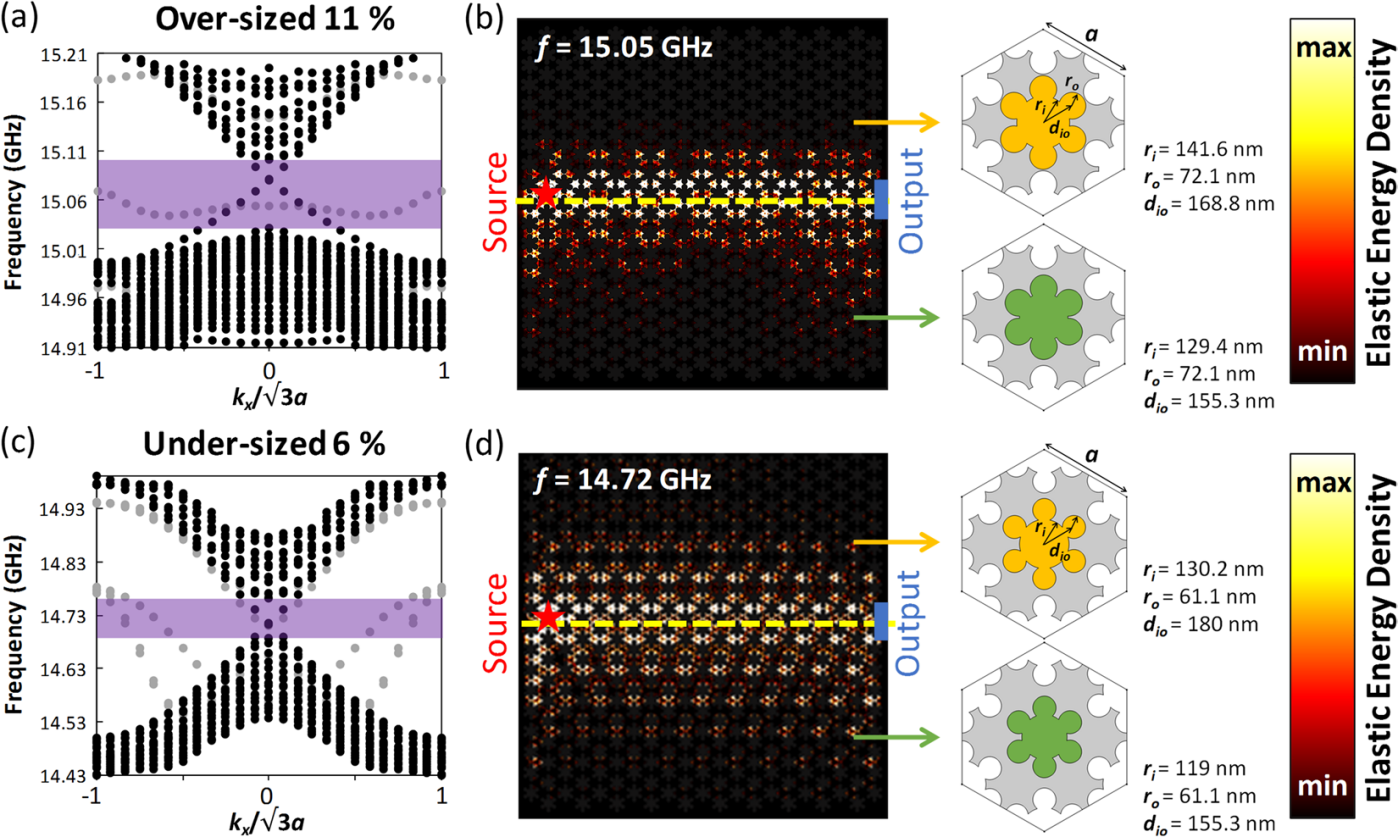
(**a**) Projected bulk band structure calculated for a supercell (11 unit cells with 6 trivial ones on top and 5 non-trivial ones on bottom of the interface) consisting of six-petal holes over-sized by 7.2 nm (an 11% increase of hole diameters with respect to ***r***_***o***_). (**b**) Full-field simulation of elastic wave transmission excited at 15.05 GHz with six-petal holes over-sized by 11%. The over-sizing errors is assumed to be uniformly distributed over the entire simulation domain. (**c**) Projected bulk band structure for a supercell consisting of six-petal holes under-sized by 4 nm (a 6% decrease of hole diameters with respect to ***r***_***o***_). (**d**) Full-field simulation of elastic wave transmission excited at 14.72 GHz with six-petal holes under-sized by 6%. Again, the under-sizing error is assumed to be uniformly distributed. The hole dimensions suffering sizing errors are given in the schematics. The yellow and green correspond to trivial and non-trivial unit cells.

To demonstrate the robustness of six-petal holey silicon to geometric variations and potential fabrication imperfections, we study the possibility of sustaining the topologically protected elastic wave transmission against sizing errors of holes, and the analysis shows that the six-petal structure offers high transmission for sizing errors up to 11% and −6%, respectively. The over-/under-sizing is simulated by increasing or decreasing the diameters of central (***r***_***i***_) and corner circles (***r***_***o***_) by same thicknesses while maintaining the distance (***d***_***io***_) untouched based on the precisely-sized hole dimensions (Fig. 2). The simulation results of supercells in Fig. 5(a,c) show that the six-petal structure allows an over-sizing up to 7.2 nm or an under-sizing down to 4 nm, which are equivalent to an 11% increase or a 6% decrease of hole sizes with respect to ***r***_***o***_. Previous work regarding silicon nanoporous structures has achieved high-precision control on the fabrication inaccuracy down to ±2 nm^40^, which is well within the allowed range of sizing errors for the six-petal design. The topological edge states, indicated by two linear dispersion curves crossing at *k*_*x*_ of 0, can be clearly observed inside bulk bandgaps (marked by light-purple boxes). We notice that the frequency corresponding to the double Dirac cones in both over- and under-sizing cases deviate from that of precisely-sizing case (Fig. 2). And we could attribute these frequency shifts to the excessive or inadequate removal of materials resulted from potential fabrication-induced errors such as those in etchings or lithographical expose. The full-field simulations in Figure (b) and (d) demonstrate robust elastic wave transmissions up to 96.1% for over-sizing of 11% and under-sizing of 6% assuming the sizing errors are uniformly distributed. The high elastic energy density confined in the vicinity of the interface, which quickly decays into the bulk regions, implies the elastic wave propagation is topologically protected. Though our simulation results in Fig. 5(b,d) consider uniform distribution of sizing errors over the entire domain, in practical fabrication, the errors of varying percentages could randomly locate. For example, majority of holes in a domain might be over-sized by 2% while a few holes might be over-sized by 6%. The 6% over-sized holes could distribute randomly either near the interface or in the bulk regions. While the topological edge states and hence topologically protected elastic wave propagation could still be supported even when the randomness is present, the transmission would be impacted and further investigation would be desired (See Supplementary Information Note 3).

## Conclusions

This work presents a holey silicon-based phononic topological insulator design that can demonstrate the topological states and the directional control of in-plane elastic waves up to 14.83 GHz when the periodicity of the unit cell is scaled to 866 nm. The six-petal design allows the *C*_6_ symmetry to form a double Dirac cone based on zone-folding and simple geometric control to break the discrete translational symmetry to achieve topological phase transition. This phase transmission can be easily shifted to low- and high-frequency by scaling up and down the periodicity. The simulations show robust elastic wave propagation with a transmission ratio up to 90% even in the presence of geometrical defects including a cavity, a disorder, and a zigzag domain wall with sharp bends. The six-petal design intrinsically avoids the potential rounding effect of sharp geometric features in fabrication which may deteriorate the performance of topological insulators. The design is also robust against the potential fabrication-induced errors such as under-sizing and over-sizing up to 6% and 11%, respectively; in both cases, we observe a shift of bandgap compared with that of the precisely-sized geometry and low-loss elastic transmission (up to 90%). These findings are promising for developing high-frequency phononic topological insulators and phononic waveguides and realizing large-scale phononic circuits for information processing.

## Methods

### Numerical simulations

Throughout this paper, the numerical simulations are performed by using the commercial Finite Element Analysis software COMSOL Multiphysics. Figures 2 and 3(a–f) are computed using the eigenfrequency study in the solid mechanics module. The Bloch periodic boundary conditions are imposed on the boundaries of the unit cell. Figures 3(g), 4 and 5 are computed by using the frequency domain study in the solid mechanics module. Low-reflection boundary conditions are imposed on the boundaries of the simulation domain to avoid undesired elastic wave leakage.

## Supplementary information

Supplementary Information

## Data Availability

The data supporting the findings in this paper are available from the corresponding author upon reasonable request.
