# Supplementary material for: Phononic topological insulators based on six-petal holey silicon structures
Source: Sci Rep. 2019 Feb 12;9:1805. doi: 10.1038/s41598-018-38387-5 (PMC6372790; doi:10.1038/s41598-018-38387-5)
Supplement: Supplementary file 1 — Supplementary Information [file 41598_2018_38387_MOESM1_ESM.docx]

**Supplementary Information**

**Phononic topological insulators based on six-petal holey silicon structures**

**Ziqi Yu, Zongqing Ren, Jaeho Lee***

Mechanical and Aerospace Engineering, University of California, Irvine, Irvine, 92697, USA

Correspondence and requests for materials should be addressed to J.L. (email: jaeholee@uci.edu)

**Note 1: Scalability of frequency supporting topological phase transition**

The frequency of the double Dirac cone and the frequency range of topologically protected band gaps demonstrated in Fig. 2 in the main texts can be scaled proportionally with the size of the geometry. In Fig. S1, if we increase the periodicity *a* from 866 (500√3) nm to 8660 (5000√3) nm, we can observe a 10-time decrease in the frequency from 14.83 GHz to 1.483 GHz, at which the phononic band structure shows the band inversion. This linear relation between the frequency and size of the geometry offers us a great flexibility of matching the required experimental condition. Moreover, it offers a quick estimation of the frequency range of edge states for the geometry of topological insulators of interest.


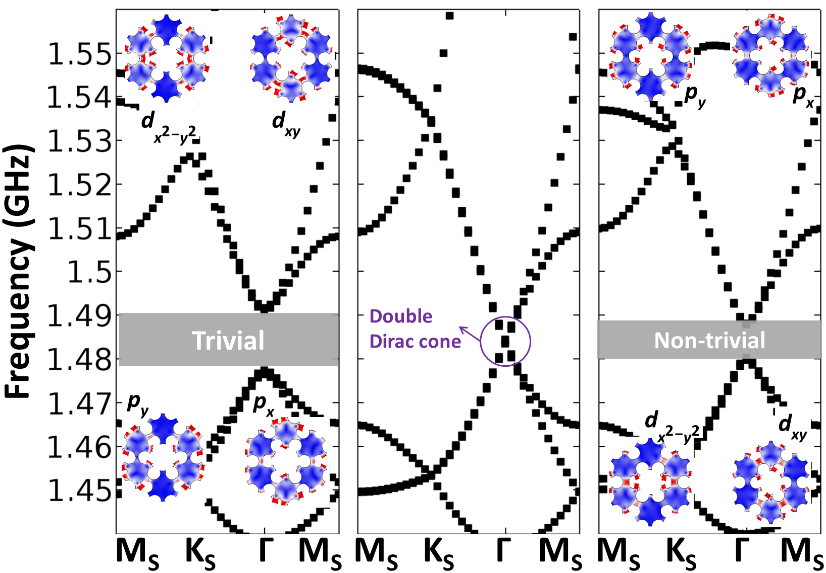


FIG. S1. The band structure calculated for the geometries scaled up by 10-time compared with the ones shown in Fig. 2 in the main texts. A double Dirac cone is formed at a frequency of *f’* = 1.483 GHz, which is 10-time smaller. By modifying the geometrical parameters, we demonstrate the opening of topologically protected band gaps around *f’*. The inverted degenerate bands at both edges of the band gaps indicate the band inversion, with the mode shapes labeled as quadruples and dipoles shown in the insets.

**Note 2: Elastic wave propagation in ordinary waveguides**

To show the robustness of topologically protected elastic wave propagation achieved by our six-petal holey silicon topological insulator, we also simulate the ordinary phononic waveguide. The ordinary phononic waveguide is constructed by involving only the trivial phononic crystals (PnCs) discussed in the main text and removing the holes along the domain wall. As shown in Fig. S2, the transmission results for three geometrical defects (similar to the ones shown in the main text) are significantly different. For the cavity and lattice disorder cases, we can clearly observe strong elastic resonances at the defects leading to dramatically inhibited elastic wave propagation afterwards; for the zigzag domain wall having two sharp bends, the elastic wave gets significantly backscattered when running into the bends, which causes a much decreased transmission.


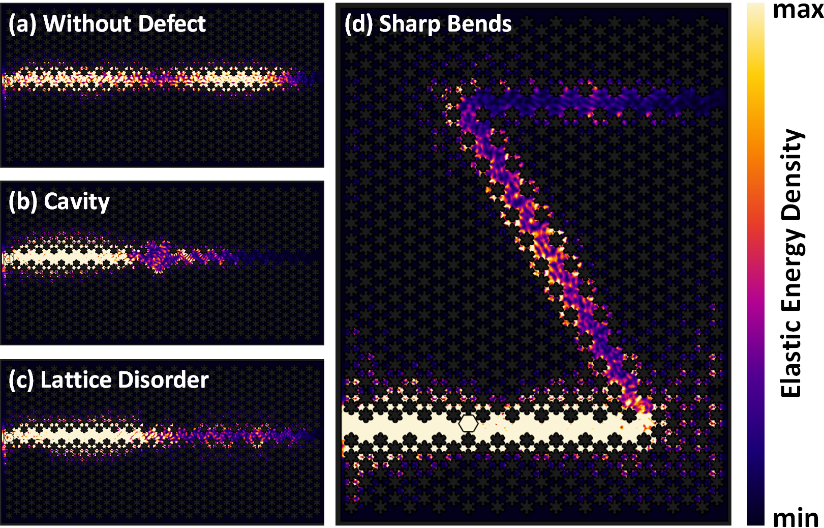


FIG. S2. Ordinary elastic waveguide constructed by having trivial phononic crystals (PnCs) in the main text and removing the holes along the domain wall. Simulated distribution of elastic energy density for the ordinary waveguide (a) without any geometrical defect, (b) with a cavity formed by filling several holes, (c) with lattice disorder by swapping holes on the edge of two domains, (d) with a zigzag domain wall having two sharp bends. We can observe large backscattering in all four cases with ordinary insulators which is fundamentally different from what we see from topological insulators with low-loss transmission in the main text.

**Note 3: Topologically protected elastic wave propagation in the presence of randomly distributed sizing errors**

To show that the helical edge state can still be supported even when randomly distributed etching errors are present, we run full-field simulations containing 367 holes with 2 % over-sized holes being as the majority and 6 % over-sized holes being randomly located along the interface or in the bulk domains. Since the helical edge state is confined in the vicinity of the interface, we expect the 6 % over-sized holes placed at/near the interface to impact more the transmission of the elastic wave. As we can see in Figure S3(a), when 2 % over-sizing errors are uniformly distributed over the entire domain, we observe 92 % elastic wave transmission from the source to the output. When four 6 % over-sized six-petal holes are randomly distributed at the interface, we can see in Figure S3(b) that the elastic wave propagation weakens when encountering the 6 % over-sized holes, but a fraction of the wave can still circumvent and reach the output, leading to a decreased transmission of 37.8 %. If we add another four 6 % over-sized holes base on the four in Figure S3(b) and place them randomly in the bulk regions, we can observe in Figure S3(c) that the transmission further deteriorates, and much less elastic wave can detour the 6 % over-sized holes to arrive at the output, resulting in a transmission only of 34 %. We notice that the effect of adding random 6 % over-sized holes in the bulk regions does not affect the transmission at the interface significantly (a 3.8 % difference in transmission). This indicates that the robustness of our design against randomly distributed etching errors is more sensitive to the randomness induced near the interface. Finally, if we gather the eight 6 % over-sized holes in Figure S3(c) at the interface, as shown in Figure S3(d), we find that the transmission of elastic wave significantly decreases when encountering the randomly distributed 6 % over-sized holes at the interface with only a small fraction detouring those holes and reaching the output. The corresponding transmission drops to 11.3 %. The reason why random distribution of 6 % over-sized holes inside the matrix of 2 % over-sized holes is the mismatched bandgaps in the band structure. As shown in Figure S4, both over-sized holes support the band inversion. However, the bandgap which will allow the existence of helical edge states for these hole dimensions do not overlap, with the lower bound of bandgap for the 2 % over-sized hole being above 14.9 GHz while the upper bound of the bandgap for the 6 % over-sized hole being below 14.9 GHz. Therefore, when 6 % over-sized holes and 2 % over-sized holes co-exist at the interface and wave is excited at 14.86 GHz, which should allow propagation in the 2 % over-sized holes, the elastic wave cannot propagate in the 6 % over-sized holes at this frequency. A hypothesis we made is that if the different over-sized holes share a frequency range over which the edge state is supported, the transmission of elastic wave, compared with the above case, could be improved. To test this, we replace the 2 % and 6 % over-sized holes with 11 % and 10 % over-sized holes and keep all other geometries untouched. In Figure S5, we first show the band structures of supercells consisting of six-petal holes over-sized by 11 % and 10 %, respectively. The edge states, indicated by straight lines crossing at *k_x_* of 0, embeds inside the bulk bandgaps; the extra bands inside the bulk bandgaps are due to the breakage of symmetry at physical boundaries of the simulation domain, which is irrelevant to the topological edge states. To calculate the elastic wave transmission, we do full-field simulations with 2 % and 6 % over-sized holes in Figure S3 replaced by 11 % and 10 %. The positions of the 10 % over-sized holes are identical to those of 6 % over-sized holes. Exciting the elastic wave at 15.05 GHz, we can see that if the six-petal holes are uniformly over-sized by 11 %, the elastic wave transmission from the source to output reaches 96.1 % (Figure S5(c)). If four 10 % over-sized holes randomly replace the 11 % holes at the interface, the transmission drops to 55.6 % (Figure S5(d)). With four more 10 % holes randomly distributed in the bulk regions, as shown in Figure S5(e), the transmission is not significantly affected, similar with what has been observed in Figure S3(c). When all eight 10 % over-sized holes concentrate to the interface, the reduction of transmission becomes significant, decreasing to 46.1 %. All transmission values enhance compared with the cases where 2 % and 6 % over-sized holes present.


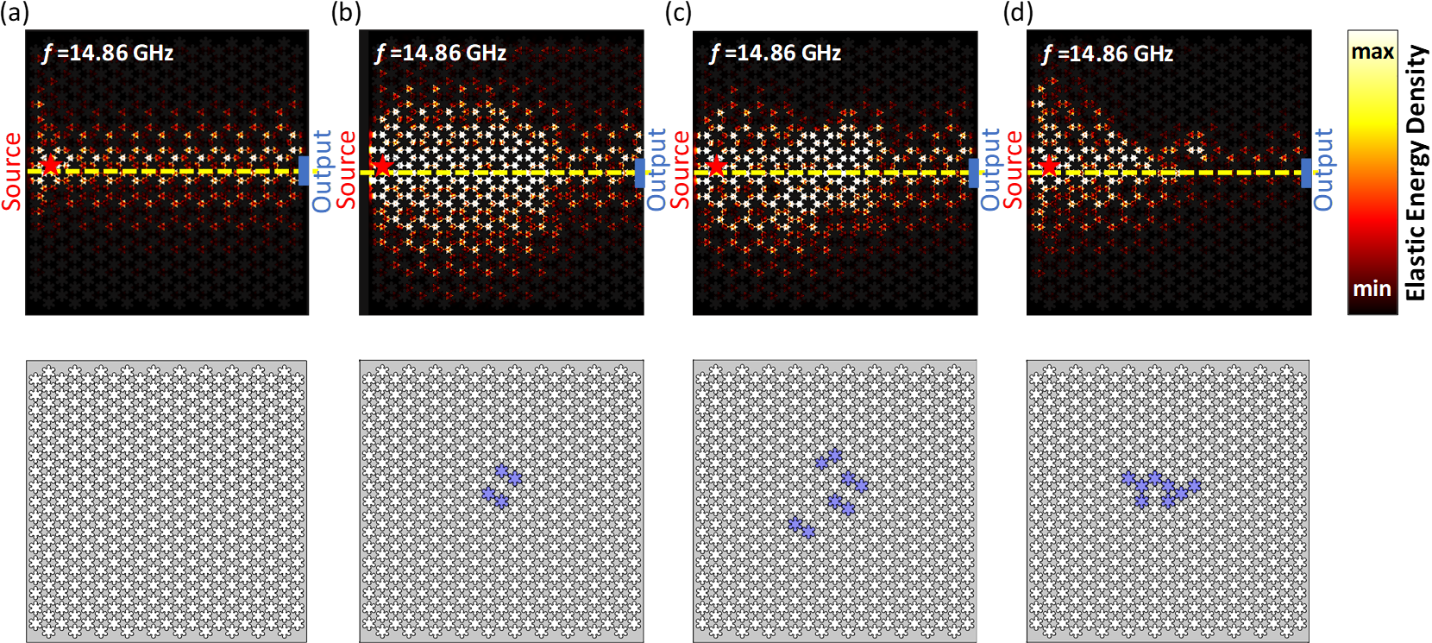


Figure S3. Full-field simulations of the elastic wave transmission in phononic topological insulator with (a) all six-petal holes over-sized uniformly by 2 %, (b) 363 of six-petal holes over-sized by 2 % and 4 holes at the interface over-size by 6 %, (c) 359 of six-petal holes over-sized by 2 % and 8 holes over-sized by 6 % randomly distributed at the interface and into the bulk region, and (d) 359 of six-petal holes over-sized by 2 % and 8 holes randomly distributed at the interface over-sized by 6 %. The transmission for the 2 % uniformly over-sized case is 92 %. When 4 holes are over-sized by 6 %, the transmission decreases to 37.8 %. When 8 holes with 6 % over-sizing are randomly located at the interface and in the bulk region, the transmission further reduces to 34 %. When 8 holes with 6 % over-sizing are randomly distributed at the interface, the elastic wave propagation transmission drops to 11.3 %.


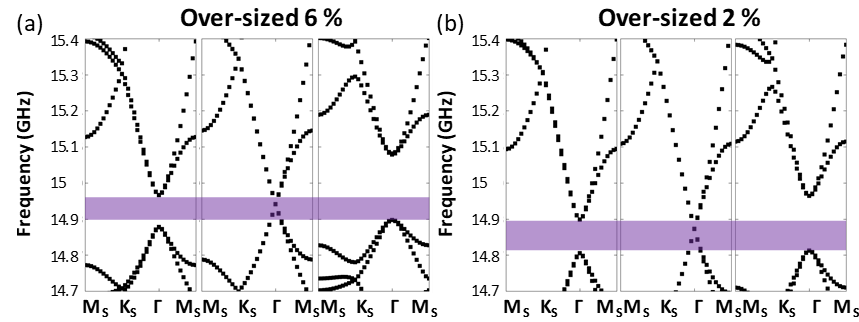


Figure S4. The phononic band structures of the six-petal holey silicon over-sized by (a) 6 % and (b) 2 %. The frequencies of bandgaps mismatch and thus the helical edge states for these cases do not have a shared frequency range.


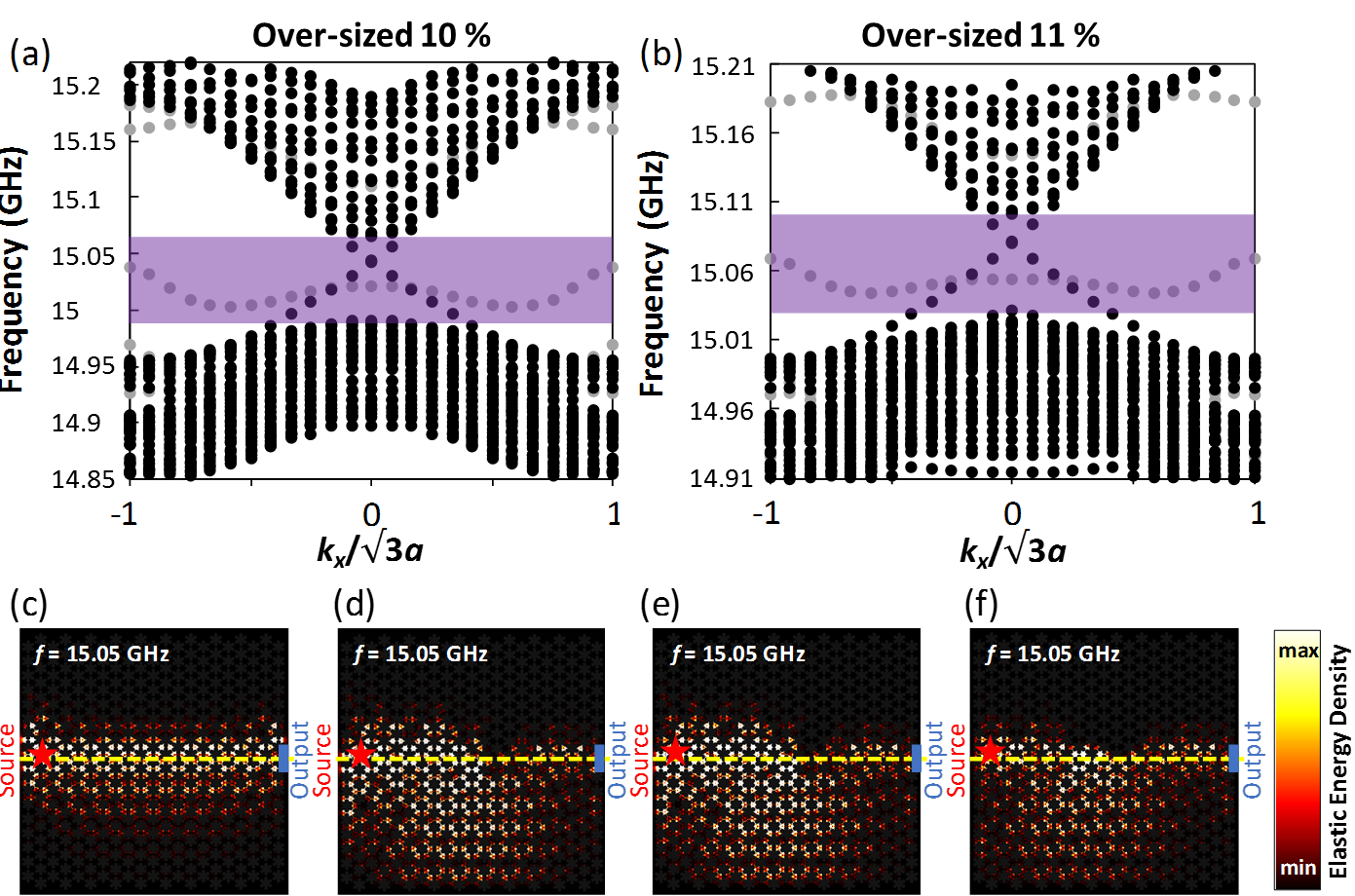


Figure S5. The band structure of supercells with six-petal holes over-sized by (a) 10 % and (b) 11 %. The bulk bandgaps are marked by light-purple boxes. The topologically protected edge states are shown by two straight lines crossing at *k_x_* of 0. The light-grey bands are confined at the physical boundaries due to the breakage of symmetry and is irrelevant to the topological edge states. The full-field simulation of elastic wave propagation shows that (c) when the six-petal holes are uniformly over-sized by 11 %, the elastic wave transmission from the source to the output reaches 96.1 %. However, when (d) four 11 % over-sized holes are replaced by 10 % over-sized holes at the interface, the transmission drops to 55.6 %. If (e) four more 10 % over-sized holes are randomly added to the bulk regions, the transmission is not significantly affected, slightly decreasing to 54 %. When (f) eight 10 % over-sized holes concentrate at along the interface, the transmission is reduced much significantly than in (d) and (e), arriving at 46.1 %. Comparing with the cases with 2 % and 6 % over-sized holes, the transmission of the field with 11 % and 10 % over-sized holes enhances.

**Note 4: Phononic band structures of over-sized and under-sized six-petal holes**

In Figure S6 and S7, we document the band structures of six-petal holes over-sized by 1 % to 10 % and under-sized by 1 % to 5 %, respectively. In all cases, we can observe that the double Dirac cones stay within overlapped bandgaps (marked by light-purple boxes). The frequency of double Dirac cone shifts from that of precisely-sized case and this could be attributed to the excessive or inadequate removal of materials associated to over- or under-sizing errors.


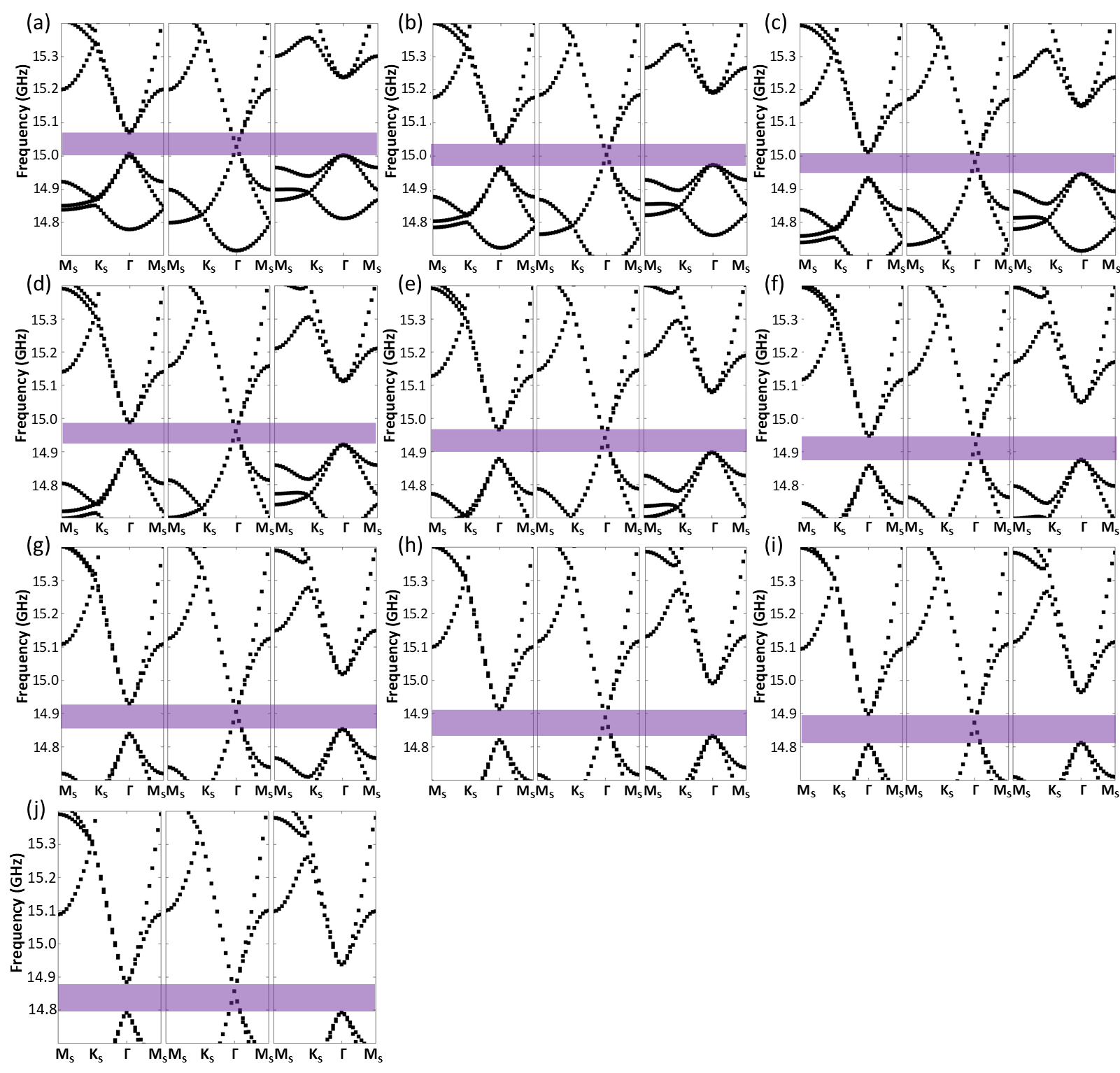


Figure S6 (a) – (i) The phononic band structure for the six-petal holes over-sized by 10 % to 1 %, where over-sizing is considered by enlarging all circles in each six-petal hole by 6.5 nm to 0.65 nm. As the over-sizing increases, the band inversion remains whereas the double Dirac cone and the overlapped bandgaps (light purple boxes) shift up with increasing material loss (i.e., less mass of the structure).


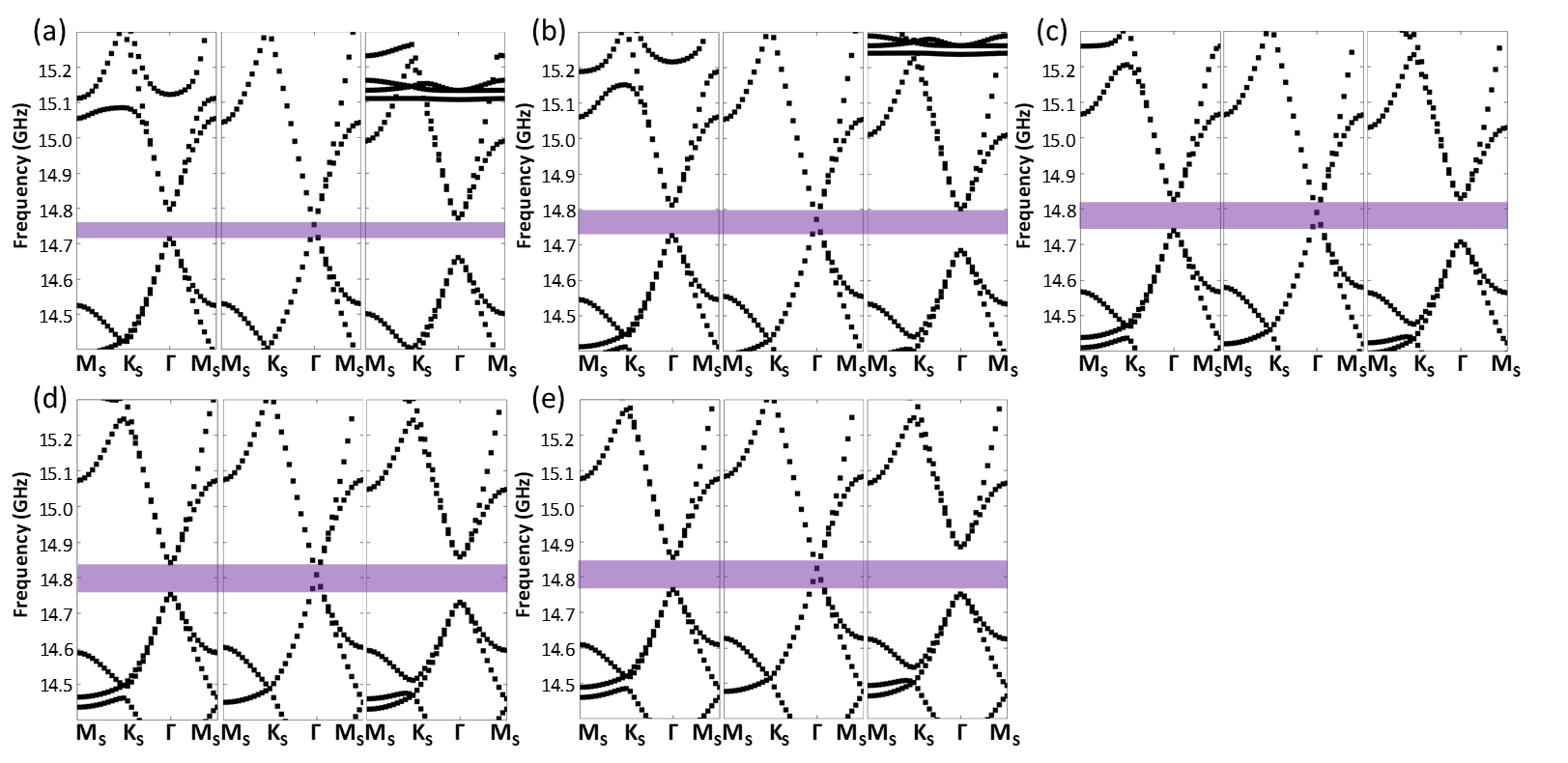


Figure S7. (a) – (e) The phononic band structure for the six-petal holes under-sized by 1 % to 5 %, where under-sizing is considered by shrinking all circles in each six-petal hole by 0.65 nm to 3.9 nm. As the under-sizing increases, the band inversion remains whereas the double Dirac cone and the overlapped bandgaps (light purple boxes) shift down with decreasing material loss (i.e., less mass of the structure).

**Note 5: Comparison of single- and multi-point excitations and one-way elastic wave propagation**

Regarding the excitation we used in the manuscript, apart from launching the wave travelling along the interface with geometric defects as shown in Figure 4 and 5 in the paper, it is also significantly useful for exciting one-way elastic wave. In Figure S8 (a) and (b), we show that by carefully engineering the amplitudes and phases of point sources, we can selectively excite left- or right-moving waves. The amplitudes and phases are based on the supercell analysis of the same hole dimensions. In (c), we show that the wave will go in both directions if we use the same amplitudes and phases as in the manuscript, which are not configured for one-way wave transport. Though waves in two directions are excited simultaneously, the transmission will not be affected because the excitation sources locate close to left boundary of the simulation domain where the low-reflection boundary condition ensures the absorption of the left-moving wave.


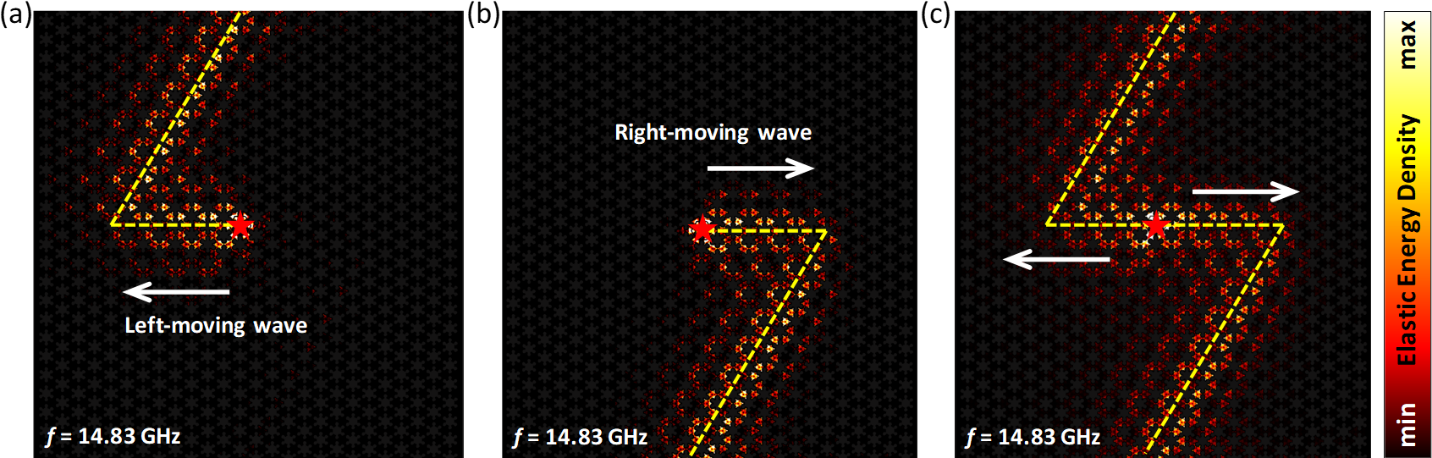


Figure S8. Full-field simulations of (a) Left-moving elastic wave excited by applying multi-point excitations with carefully configured amplitudes and phases at the position marked by the red star. (b) Right-moving elastic wave launched by applying multi-point excitations with the same amplitudes as in (a) but opposite phases. (c) Elastic waves excited to propagate in both directions by using the same three-point excitation scheme (same force amplitudes and phases) as in the manuscript. The holes are precisely-sized.
